# Meta-analysis of the success rate of heartbeat recovery in patients with prehospital cardiac arrest in the past 40 years in China

**DOI:** 10.1186/s40779-020-00263-7

**Published:** 2020-07-07

**Authors:** Xiang-Min Gu, Shi-Bin Yao, Zhong-jie He, Yong-Gang Wang, Zhi-Hui Li

**Affiliations:** 1 Tianjin Binhai New Area Center for Disease Control and Prevention, Tianjin, 300450 China; 2 Emergency Department, Affiliated Hospital of Chifeng College, Chifeng, 024005 Inner Mongolia China; 3 The Fourth Medical Center of Chinese PLA General Hospital, Beijing, 100048 China; 4 Beijing Platinum Ten Minutes Time-effect Emergency Technology Research Academy, Beijing, 100039 China; 5 Beijing Chaoyang District Jiangtai Community Health Service Center, Beijing, 100016 China

**Keywords:** Sudden cardiac arrest, Cardiopulmonary resuscitation, Peri-cardiac arrest period, Platinum 10 min, Meta-analysis

## Abstract

**Background:**

Systematic evaluation of the successful heartbeat recovery rate (HRR) in patients during the platinum ten minutes after cardiac arrest.

**Methods:**

The databases of CNKI (January 1979–March 2019), Chongqing VIP (January 1989–March 2019), Wanfang (January 1990–March 2019) and Web of Science (1900-May 2020) were searched. To collect the clinical data of patients with cardiac arrest before hospitalization and analyze the cardiopulmonary resuscitation (CPR) at different times. Literature selection and data extraction were carried out by two researchers independently, and the meta package of R software (version 3. 61) was used for analysis.

**Results:**

A total of 116 papers met the inclusion criteria, including 37,181 patients. Of these patients, 3367 had their heartbeats successfully restored. The results showed a high degree of heterogeneity (*χ*^2^ = 6999.21, *P* < 0.01, I^2^ = 97.6%). The meta-analysis was conducted using a random-effects model. The combined effect size was 0.199 (0.157–0.250). (1) According to the five CPR groups (International Cardiopulmonary Resuscitation Guide 2000, 2005, 2010, 2015 and other versions), the HRR of other versions [0.264 (0.176–0.375)] was higher than the International Cardiopulmonary Resuscitation 2005 edition [0.121 (0.092–0.158)]. (2) The rescue time was divided into the 0 to ≤5 min group, the 5 to ≤10 min group, the 10 to ≤15 min group, and the > 15 min group. The HRR were 0.417 (0.341–0.496), 0.143 (0.104–0.193), 0.049 (0.034–0.069), and 0.022 (0.009–0.051), respectively. The HRR was higher in the 0 to ≤5 min group than in the 5 to ≤10 min group, the 10 to ≤15 min group and the > 15 min group. There was no difference between the 10 to ≤15 min group and the > 15 min group. (3) When the groups were stratified with the cutoff of 10 min, the ≤10 min group HRR [0.250 (0.202–0.306)] was higher than the > 10 min group rate [0.041 (0.029–0.057)]. (4) The HRR of the telephone guidance group was [0.273 (0.227–0.325)] lower than that of the 0 to ≤5 min group [0.429 (0.347–0.516)] but higher than that of the 5 to ≤10 min group, the 10 to ≤15 min group, and the > 15 min group. (5) The HRR of the witness group [0.325 (0.216–0.458)] was not different from that of the 0 to ≤5 min group, but it was higher than those of the 5 to ≤10 min group, the 10 to ≤15 min group and the > 15 min group. (6) There was no significant difference HRR between the witnessed group, the telephone guidance group and the ≤10 min group.

**Conclusions:**

(1) The HRR is time-sensitive, and early rescue can improve it. (2) CPR performed within the platinum ten minutes must be executed by the public, and other forces are auxiliary. (3) The concept of peri-cardiac arrest period (PCAP) should be established and improved to guide CPR.

## Background

Cardiopulmonary resuscitation (CPR) is an important measure used to restore a heartbeat in patients with cardiopulmonary arrest. It is very useful for gaining treatment time and saving patients’ lives. The number of cardiac arrests in the world is as high as 800–900 million per year [1], and approximately 70% of patients experience cardiac arrest before arriving at a hospital. The number of sudden deaths in China is 1 million per year [2], but the rate of prehospital heartbeat recovery in out-of-hospital cardiac arrest patients is only 17.1% [3], which is lower than in developed countries. Thus, the author proposes the concepts of “peri-sudden death period (PSDP)” and “peri-cardiac arrest period (PCAP)” to guide the public in preventing and responding to cardiopulmonary arrest [4]. In first-aid, the platinum 10 min refers to the time-saving principle and first-aid theory of treatment after accidental injury; in the first 10 min, the professionals cannot reach the patient, but it is the most critical time to receive treatment. This stage is the key link with the “120″ emergency system, and it includes CPR, remove airway foreign body, defibrillation, and stopping bleeding. It is necessary to propagate the first-aid knowledge and skills such as airway asphyxia from the start of the witness. The PSDP is the most important part of the study. The National Self-help and Rescue Volunteer Service Alliance conducts a meta-analysis of the success rate of heartbeat recovery in patients with prehospital cardiac arrest in China every 5 years to assess and improve the status of prehospital or public CPR.

## Methods

### Inclusion and exclusion criteria

CPR patients rescued by different international versions of guidelines. Study type: Retrospective case summary and analysis; Language limit: Chinese; Study object: CPR patients with cardiac arrest caused by various diseases before hospital; patients with CPR have definite time in different stages and CPR recovery. The results showed that the patients had spontaneous rhythm and pulse recovery. Exclusion criteria: Literature exclusion of patients without event occurrence time, repetition, unreported data and number of patients ≤10.

### Retrieval strategy

The computer searches the CNKI (January 1979–March 2019), Wanfang database (January 1990–March 2019), Chongqing VIP database (January 1989–March 2019), and Web of Science (1900-May 2020). The search terms are title, title or theme, including “心跳骤停+院前+中国(cardiac arrest + out of hospital + China)”, and“心肺复苏+院前+中国 (CPR +out of hospital+ 中国)”.

### Paper screening, data extraction, and quality evaluation

Select the documents according to the pre-determined inclusion and exclusion labels. Two researchers read the titles and abstracts of the obtained documents independently. After excluding the documents that clearly do not meet the inclusion criteria, further read the full text of the documents that may meet the inclusion criteria to determine whether they meet the inclusion criteria. In case of any disagreement, it shall be discussed or submitted to a third party for adjudication. The contents of the literature must include: (1) general information: title, author, date of publication and source of the literature; (2) research features: whether there is CPR rescue time and rescue has a clear number of people and deaths; (3) clinical outcome indicators include one of the following indicators: Patients have pulse recovery, spontaneous breathing recovery, consciousness recovery and discharge data.

### Statistical analyses

Analyses were conducted using R Meta package software (version 3.61). The *χ*^2^ test was used to test the heterogeneity of the data. *P* > 0.05 denoted no significant heterogeneity in the dependent variables. A meta-analysis was conducted using a fixed-effects model. *P* < 0.05 denoted heterogeneity in the dependent variables. A random-effects model can be used when heterogeneity remains after controlling for it and the combined data continue to have clinical significance [5]. The *I*^2^ statistic was used to reflect the magnitude of the heterogeneity of the combined effect. Larger *I*^2^ values denote greater heterogeneity. If *I*^2^ < 25%, then mild heterogeneity exists across the studies. If 25% < *I*^2^ < 75%, then moderate heterogeneity exists across the studies. If *I*^2^ > 75%, then a high degree of heterogeneity exists across the studies. A funnel plot was used to estimate the publication bias. A two-sided test was used when a need existed to test the above statistical analyses. The significance level was set at 0.05.

## Results

### Literature retrieval

**Fig. 1:**
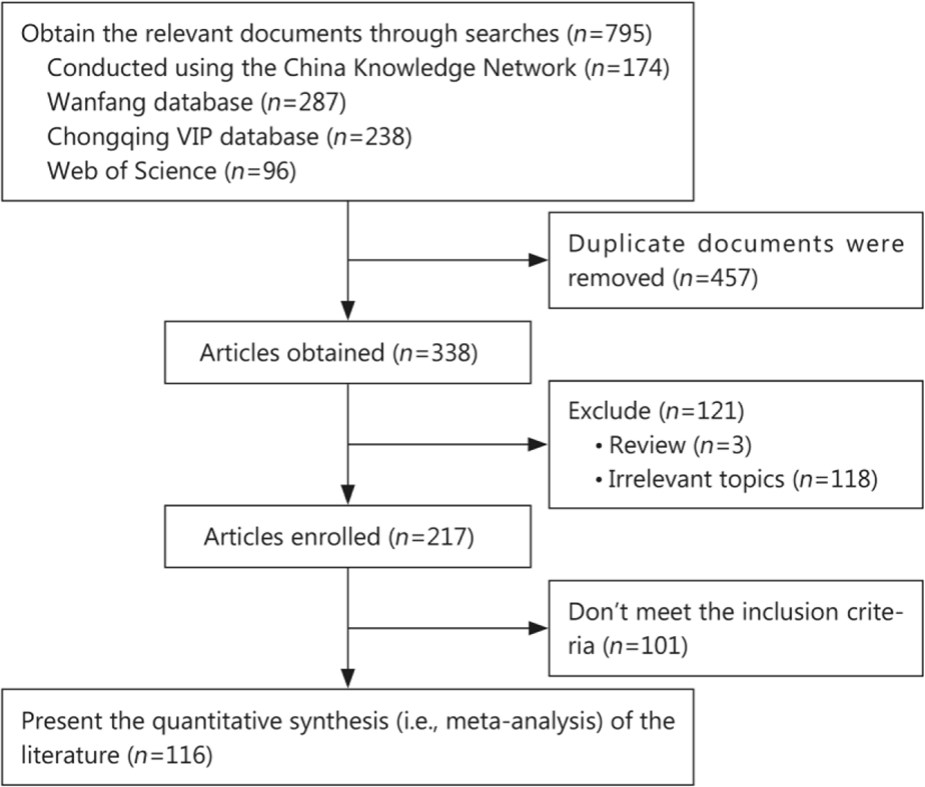
Flowchart of literature screening process

A total of 699 relevant papers were initially retrieved. After screening the papers, 116 papers were included in the final analysis [6–121]. A total of 37,181 patients were included in these 116 papers. Figure 1 shows a flow chart of the literature screening process.

### General information of the papers

**Fig. 2:**
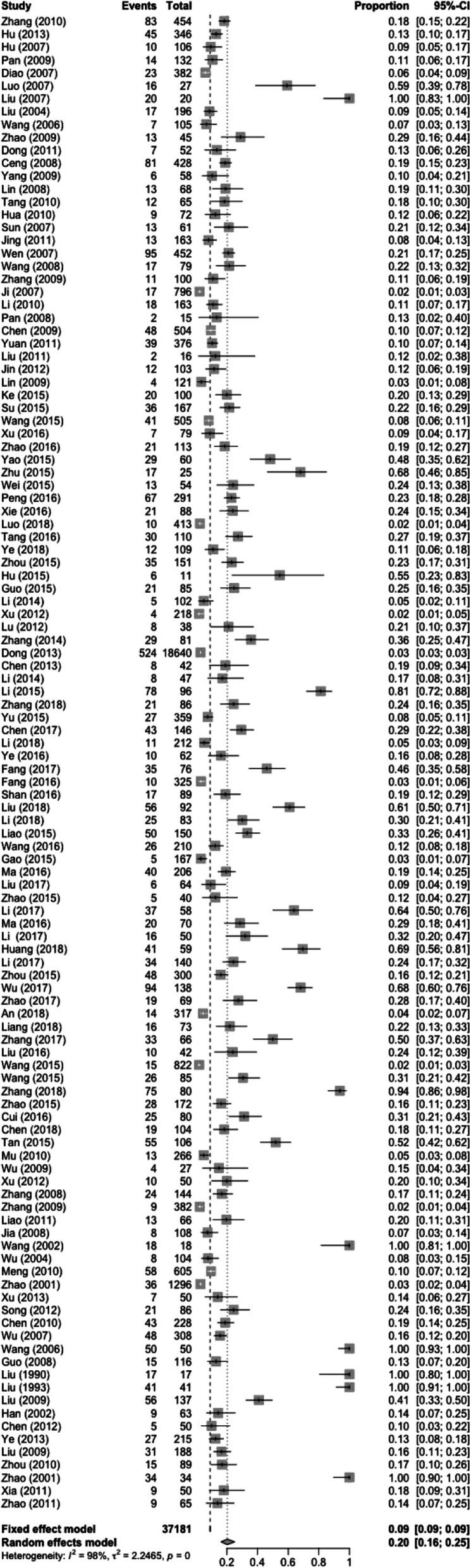
CPR success rates

**Fig. 3:**
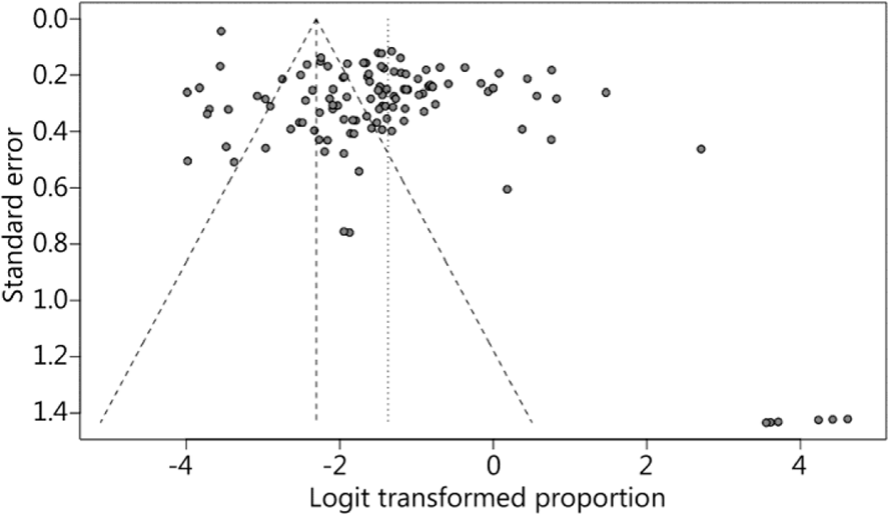
Funnel plot of the publication bias

In total, 116 papers met the inclusion criteria, containing 37,181 patients. Of these patients, the heartbeats of 3367 patients were restored. The results of the heterogeneity test (*χ*^2^ = 6999.21, *P* < 0.01, I^2^ = 97.6%) indicated a high degree of heterogeneity. A meta-analysis was conducted using a random-effects model. The combined effect size was 0.199 (0.157–0.250; Fig. 2). The publication bias test was performed with the Egger test (t = 1.3718, *p* = 0.1728). The results showed that the articles did not have publication bias (Fig. 3).

### CPR was performed using the methods provided by different guidelines

**Fig. 4:**
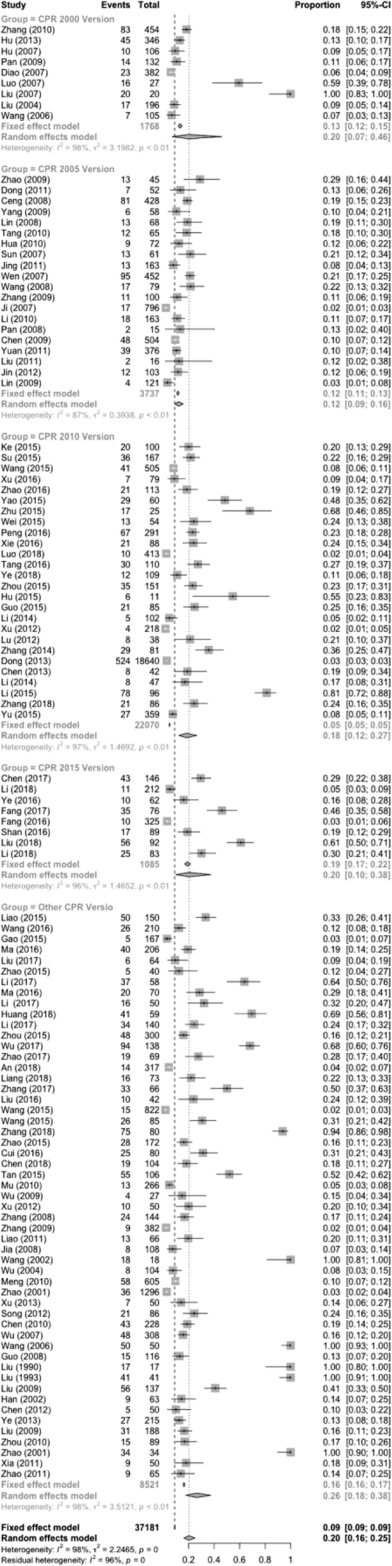
Comparisons of the HRR using the methods provided in the different guidelines

The different rescue programs used in the included studies were the International Cardiopulmonary Resuscitation Guide 2000、2005、2010、2015 Edition and other guidelines. Among these, The International Cardiopulmonary Resuscitation 2000 edition was used in 9 articles, 2005 edition was used in 20 articles, 2010 edition was used in 26 articles, 2015 edition was used in 8 articles, and other guidelines were used in 53 articles. The results show that HRR [0.121 (0.092–0.158)] when the 2005 guidelines were used was significantly different from that when other versions of the guidelines were used [0.264 (0.176–0.375)]. There was no significant difference with other CPR versions (as shown in Fig. 4). Since the literatures included in other editions have reported more successful heartbeat recovery results, the results of the 2005 edition included in the publication bias were less, resulting in differences in the HRR. There may not be a difference. The HRR is not related to the version of the guidelines used for CPR. Timeliness is the key to the success rate. The results are shown in Table 1.

**Table 1:** Comparisons of the HRR using the methods provided in different guidelines

Guideline	Case	Heterogeneity test			Model	Success rate	Confidence interval (95%CI)	Number of success	Number of patients
		Q-value	*P*-value	*I*^2^ (%)					
CPR 2000 Version	9	68.70	< 0.001	98.2	Random	0.202	0.071, 0.458	235	1, 768
CPR 2005 Version	20	140.06	< 0.001	87.0	Random	0.121^a^	0.092, 0.158	432	3, 737
CPR 2010 Version	26	1, 306.35	< 0.001	97.2	Random	0.169	0.104, 0.265	1, 098	22, 070
CPR 2015 Version	8	162.12	< 0.001	95.9	Random	0.204	0.098, 0.377	207	1, 085
Other CPR Version	53	992.53	< 0.001	98.2	Random	0.264	0.176, 0.375	1, 395	8, 021
Total	116	2, 669.76	< 0.001	98.2	Random	0.202	0.158, 0.252	3, 367	37, 181

^a^The difference between the CPR2005 version and other CPR version is significantly different

CPR 2000 Version: 2000 Guidelines for CPR and Emergency Cardiovascular Care.

CPR 2005 Version: 2005 Guidelines for CPR and Emergency Cardiovascular Care.

CPR 2010 Version: 2010 Guidelines for CPR and Emergency Cardiovascular Care.

CPR 2015 Version: 2015 Guidelines for CPR and Emergency Cardiovascular Care.

Other CPR Version including: 2010 American Heart Association Guidelines For Cardiopulmonary Resuscitation and Emergency, AHA Guidelines for CPR, 2005 AHA Guidelines for CPR, and Pediatric Advanced Life Support.

### Time-dependent conditions of CPR

**Fig. 5:**
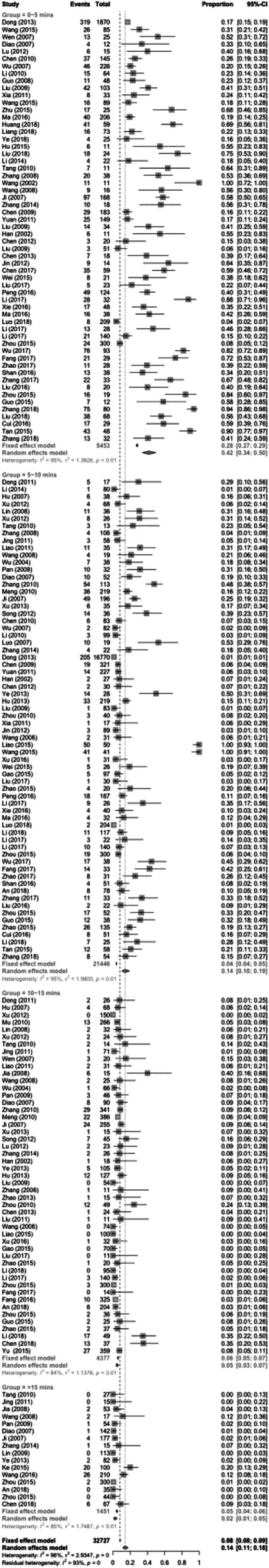
Comparison of the CPR success rates at different times

Patients were divided into the 0 to ≤5 min group, 5 to ≤10 min group, 10 to ≤15 min group, and > 15 min group according to different CPR initiation times. According to the times recorded in the studies, 58 were included in the 0 to ≤5 min group, 66 in the 5 to ≤10 min group, 49 in the 10 to ≤15 min group, and 16 in the > 15 min group. Results: The 0 to ≤5 min group HRR [0.417 (0.341–0.496)] was higher than those of the 5 to ≤10 min group [0.143 (0.104–0.193)], the 10 to ≤15 min group [0.049 (0.034–0.069)] and the > 15 min group [0.022 (0.009–0.051)]. The 5 to ≤10 min group HRR [0.143 (0.104–0.193)] was higher than those of the 10 to ≤15 min group [0.049 (0.034–0.069)] and the > 15 min group [0.022 (0.009–0.051)]. There was no different in the HRR between the 10 to ≤15 min group and the > 15 min group. The results show that with the prolongation of the duration before the initiation of CPR, the survival rate decreases. The results are shown in Fig. 5 and Table 2.

**Table 2:** Comparison of the HRR at different times

Group	Case	The test for heterogeneity			Model	Success rate	Confidence interval (95%CI)	Number of success	Number of patients
		Q-value	*P-value*	*I*^2^ (%)					
0 to ≤5 min	58	830.18	< 0.001	94.9	Random	0.417	0.341, 0.496	1, 523	5, 453
5 to ≤10 min	66	1, 538.87	< 0.001	94.8	Random	0.143^a^	0.104, 0.193	911	21, 446
10 to ≤15 min	49	153.65	< 0.001	83.8	Random	0.049^a,b^	0.034, 0.069	259	4, 377
> 15 min	16	54.28	< 0.001	84.7	Random	0.022^a,b,c^	0.009, 0.051	67	1, 451
Total	189	2, 576.98	< 0.001	96.3	Random	0.141	0.109, 0.182	2, 760	32, 727

^a^The difference between the 0 to≤5 min group and other three group CPR are significantly different

^b^The difference between the 5 to ≤10 min group, the 10 to ≤15 min group and the > 15 min group are significantly different

^c^The different between the > 15 min group, the 5 to ≤10 min group and 0 to≤5 min group are significantly different

**Fig. 6:**
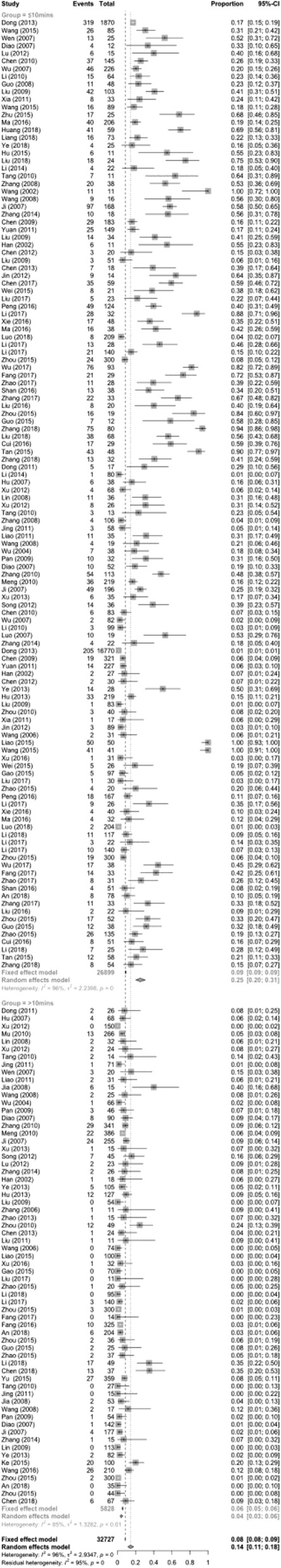
The HRR for the ≤10 min and > 10 min groups

According to the different CPR initiation times, the two groups were the ≤10 min group and the > 10 min group. According to the recorded times in the studies, 124 articles were included in the ≤10 min group, and 65 articles were in the > 10 min group. The ≤10 min group HRR [0.250 (0.202–0.306)] was higher than the > 10 min group HRR [0.041 (0.029–0.057)]. The results are shown in Fig. 6 and Table 3.

**Table 3:** The HRR in the ≤10 min and > 10 min groups

Group	Case	Heterogeneity test			Model	Success rate	Confidence intervals (95%CI)	Number of success	Number of patients
		Q-value	P-value	*I*^2^ (%)					
≤10 min	124	3, 214.97	< 0.001	96.3	Random	0.250	0.202, 0.306	2, 434	26, 899
> 10 min	65	208.58	< 0.001	85.3	Random	0.041^a^	0.029, 0.057	326	5, 828
Total	189	3, 423.55	< 0.001	95.3	Random	0.142	0.109, 0.181	2, 760	32, 727

^a^The difference between the ≤10 min group and > 10 min group is significantly different

**Fig. 7:**
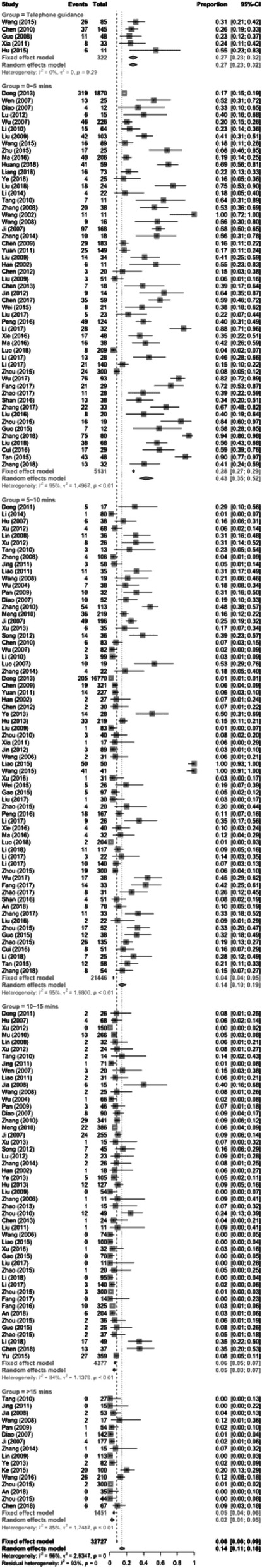
The HRR of the telephone guidance group and the other groups at different times

According to whether telephone guidance for first aid was provided, the groups in the comparative analysis were the telephone instruction group, 0 to ≤5 min group, 5 to ≤10 min group, 10 to ≤15 min group and > 15 min group. Telephone guidance was provided in 5 articles, while there were 53 in the 0 to ≤5 min group, 66 in the 5 to ≤10 min group, 49 in the 10 to ≤15 min group, and 16 in the > 15 min group. The HRR of the telephone guidance group was [0.273 (0.227–0.325)] less than that of the 0 to ≤5 min group [0.429 (0.347–0.516)] but higher than those of the 5 to ≤10 min group [0.143 (0.104–0.193)], the 10 to ≤15 min group [0.049 (0.034–0.069)] and the > 15 min group [0.022 (0.009–0.051)]. The results are shown in Table 4 and Fig. 7.

**Table 4:** The HRR of the telephone guidance group and other groups at different times

Group	Case	Heterogeneity test			Model	Success rate	Confidence interval (95%CI)	Number of success	Number of patients
		Q-value	P-value	*I*^2^ (%)					
Telephone guidance	5	4.99	< 0.001	0.0	Random	0.273	0.227; 0.325	88	322
0 to ≤5 min	53	824.93	< 0.001	95.2	Random	0.429^a^	0.347; 0.516	1, 435	5, 131
5 to ≤10 min	66	1, 538.87	< 0.001	94.8	Random	0.143^b^	0.104; 0.193	911	21, 446
10 to ≤15 min	49	153.65	< 0.001	83.8	Random	0.049^b^	0.034; 0.069	259	4, 377
> 15 min	16	54.28	< 0.001	84.7	Random	0.022^b^	0.009; 0.051	67	1, 451
Total	189	2, 576.72	< 0.001	93.4	Random	0.139	0.109; 0.183	2760	32, 727

^a^The difference between the telephone guidance group and 0 to ≤5 min is significantly different

^b^The difference between the telephone guidance group and 5 to ≤10 min group, 10 to ≤15 min group and > 15 min group are significantly different

**Fig. 8:**
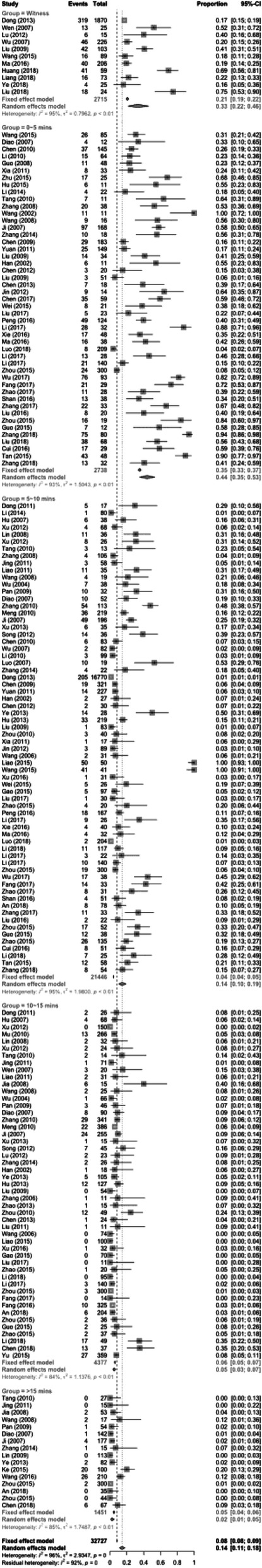
The HRR of the witness + public group and other groups at different times

According to the presence or absence of witnesses, the patients were divided into the witness + public group, the 0 to ≤5 min group, the 5 to ≤10 min group, the 10 to ≤15 min group, and the > 15 min group for a comparative analysis. There were 11 articles included in the witness + public group, 47 in the 0 to ≤5 min group, 66 in the 5 to ≤10 min group, 49 in the 10 to ≤15 min group, and 16 in the > 15 min group. Results: The HRR of the witness + public group [0.325 (0.216–0.458)] was not different from that of the 0 to ≤5 min group [0.439 (0.351–0.533)], but it was higher than those in the 5 to ≤10 min group [0.143 (0.104–0.193)], the 10 to ≤15 min group [0.049 (0.034–0.069)] and the > 15 min group [0.022 (0.009–0.051)]. The results are shown in Table 5 and Fig. 8.

**Table 5:** The HRR of the witness + public group and other groups at different times

Group	Case	The test for heterogeneity			Model	Success rate	Confidence interval (95%CI)	Number of success	Number of patients
		Q-value	P-value	I^2^ (%)					
Witness	11	140.71	< 0.001	95.0	Random	0.325	0.216; 0.458	561	2, 715
0 to ≤5 min	47	546.33	< 0.001	93.3	Random	0.439	0.351; 0.533	962	2, 738
5 to ≤10 min	66	1, 538.87	< 0.001	94.8	Random	0.143^a^	0.104; 0.193	911	21, 446
10 to ≤15 min	49	153.65	< 0.001	83.8	Random	0.049^a^	0.034; 0.069	259	4, 377
> 15 min	16	54.28	< 0.001	84.7	Random	0.022^a^	0.009; 0.051	67	1, 451
Total	189	2, 433.84	< 0.001	92.3	Random	0.139	0.108; 0.183	2760	32, 727

^a^The difference between the witness group and 5 to ≤10 min group,10 to ≤15 min group and > 15 min group are significantly different

**Fig. 9:**
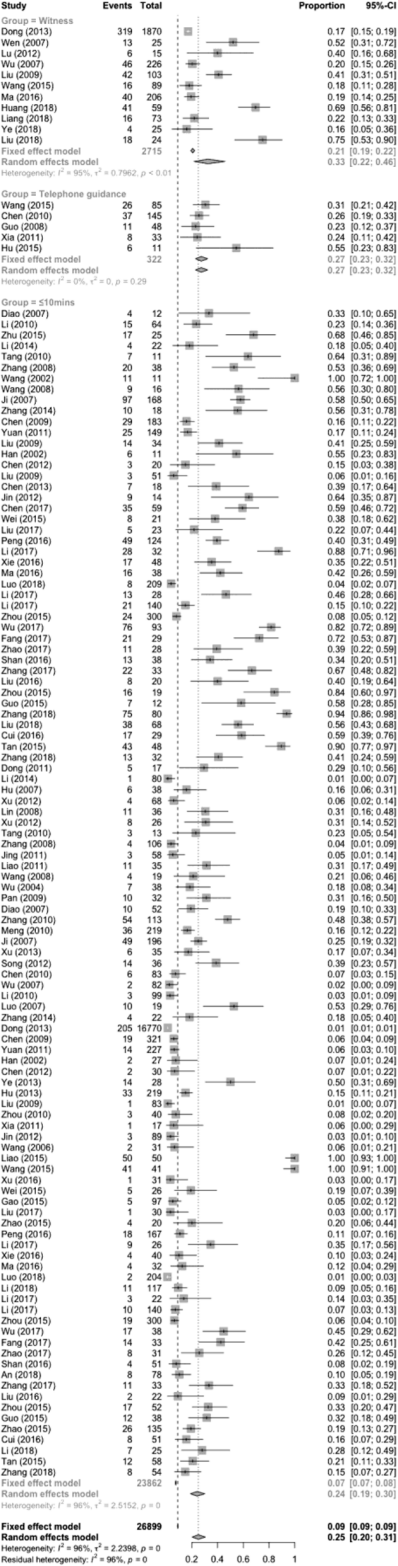
The HRR of the witness +public group, telephone guidance group and ≤ 10 min group

The witness + public group, telephone guidance group and ≤ 10 min group were compared. In total, 11 articles were included in the witness + public group, 5 in the telephone guidance group, and 108 in the ≤10 min group. The results show that the HRR of the witness + public group was 0.325 (0.216–0.458), and the HRR of the telephone guidance group was 0.273 (0.227–0.325), while the ≤10 min group HRR was 0.240 (0.188–0.302). There were no significant differences among the three groups, as shown in Table 6 and Fig. 9.

**Table 6:** The HRR of the witness + public group, telephone guidance group and ≤ 10 min group

Group	Case	Heterogeneity test			Model	Success rate	Confidence interval (95%CI)	Number of success	Number of patients
		Q-value	P-value	I^2^ (%)					
Witness	11	140.71	< 0.001	95.0	Random	0.325	0.216, 0.458	561	2, 715
Telephone guidance	5	4.99	< 0.001	0.0	Random	0.273	0.227, 0.325	88	322
≤10 min	108	3, 005.67	< 0.001	95.9	Random	0.240	0.188, 0.302	1, 785	23, 862
Total	124	3, 151.37	< 0.001	96.1	Random	0.251	0.198, 0.311	2, 434	26, 899

## Discussion

Cardiac arrest is the most critical condition in the clinic, and patients can obtain a better recovery if they receive effective CPR immediately. The overall prognosis of patients with cardiac arrest outside the hospital is currently poor. According to the American Heart Association, there were approximately 360,000 out-of-hospital cardiac arrest patients in the United States in 2016, and the discharge rate was only 10.6% [122]. The out-of-hospital survival rate of out-of-hospital cardiac arrest in the Asia-Pacific region ranges from 0.8 to 9.0% [123]. Due to the limitations of the studies in this analysis and the analysis of other aspects of CPR, we used concept of the platinum ten minutes to investigate CPR in China. Extracting the single clinical index of the earliest recovered heartbeat during CPR to analyze early CPR can reflect the state of CPR in China to some extent. There were 116 articles that met the inclusion criteria, with a total of 37,181 patients, and the number of patients with recovered heartbeats was 3367, with a heartbeat recovery success rate of 19.9%.

The limitations of this study are as follows: (1) This study included 116 articles, all of which were retrospective observational studies. The quality of the clinical data varied substantially, and it was impossible to evaluate the comprehensive cardiopulmonary cerebral resuscitation of the heartbeat, respiration, brain function and vital status. (2) The numbers of papers in the telephone guidance and the witness + public groups were small, and more studies need to be performed. (3) When fewer than 10 patients were discussed in an article, that article was excluded from this analysis. Despite these limitations, this study can still provide us with useful guidance.

Version comparison: In articles using the five different versions of the CPR guidelines (The International Cardiopulmonary Resuscitation Guide 2000, 2005, 2010, 2015 and other guidelines), the HRR rates were 20.2, 12.1, 16.9, 20.4, and 26.4%, respectively. Among the different guidelines used, the HRR was different when the 2005 edition was used than when other editions were used. Studies using editions other than the 2005 guidelines reported more success in terms of heartbeat recovery, with publication biases, while the articles using the 2005 edition showed less publication bias, resulting in differences in the HRR. The HRR has nothing to do with the guideline version of CPR, and timeliness is the key to its success rate.

### Timeliness analysis

The CPR initiation time was divided into 0 to ≤5 min, 5 to ≤10 min, 10 to ≤15, and > 15 min. The HRR were 41.7, 14.3, 4.9 and 2.2%, respectively. The 0 to ≤5 min group had a higher success rate than the other 3 groups, and the 5 to ≤10 min group had a higher success rate than the last 2 groups, while the 10 to ≤15 min group and the > 15 min group were not different from each other, indicating that the rescue survival rate decreased with the prolongation of the time to the initiation of CPR. The conclusion is the sooner CPR is started, the better [124]. For every minute CPR and defibrillation are delayed, the success rate is reduced by 7–10%. Therefore, they do not have the best aging platform, and there is no such rule of prime time [125].

### Artificial differences

The HRR of the telephone guidance group and the witness + public group were 27.3 and 32.5%, respectively, and the rate in the telephone guidance group was less than that in the 0 to ≤5 min group. There was no difference between the HRR between the witness + public group and the 0 to ≤5 min group, but both had rates that were higher than those in the last three time strata, indicating that they were superior. This is contrary to our expectations. To further support this conclusion, we need more detailed, accurate research.

### Rescuer and time-effectiveness analysis

The HRR were different among the witness group (32.5%), telephone guidance group (27.3%) and ≤ 10 min group (24.0%). This order of success rates matches our expectation, although they are not significantly different. It is possible that the numbers of studies included in the telephone guidance group and the witness group were small, and more studies with larger sample sizes should be conducted in the future. There is a significant difference in the HRR between the ≤10 min group (25.0%) and the > 10 min group (4.1%), suggesting that CPR should be performed within 10 min and that every minute counts in terms of improving the HRR; effective treatment greatly enhances the possibility of survival [126].

Public cardiopulmonary resuscitation(P-CPR) and telephone-guided CPR within the platinum ten minutes is the key to first aid. For the case of sudden cardiac arrest, it is difficult for emergency medical personnel to reach the emergency site in a short time. The best first aid can be provided by the injured person and any eyewitnesses. Timely self-help and rescue by witnesses can be valuable [127]. It may be that the number of articles included is small. We hope that the HRR of the telephone guidance group and the witness group will meet or exceed the HRR of the 0 to ≤5 min group.

In the article we published 5 years ago, the exclusion criteria were patients with repeated, unreported data and studies with fewer than 20 patients [3], while in this study, the exclusion criteria were duplicate patients, patients with unreported data and studies with fewer than 10 patients. The number of articles included in the study increased by 59, and the successful HRR increased from 17.1 to 19.9% [3], but the real-world HRR is still low. In the analysis of the different versions of the CPR guidelines, 8 articles used the 2015 edition, and the HRR was not significantly different from those in the articles using the other versions of the guidelines. Because the ≤1 min group would have had fewer articles and the time boundary was difficult to define, it was included in the 0 to ≤5 min group. Compared with the studies available 5 years ago, the number of studies in the witness + public and telephone guidance groups increased by 4 and 2, respectively, and CPR was shown to be time-sensitive. We should increase public awareness and make first aid technology available to the public. Only self-help and action on the part of witnesses can lead to a higher rescue success rate.

In previous studies, we found that in order to improve the success rate of cardiac arrest, we must change the node awareness of cardiac arrest to the process of understanding, and define the beginning and outcome of cardiac arrest as PCAP [4]. We can divide the clinical process of PCAP into three periods: pre-PCAP, mid-PCAP, and late-PCAP. When the patient has critical symptoms such as hypothermia, altered consciousness, abnormal breathing, abnormal heart rhythm, unstable blood pressure, oliguria or anuria, a hemoglobin level < 5 g, critical electrolyte values, or severe acidosis or when the patient shows further deterioration of the aforementioned conditions/symptoms and signs and is on the verge of respiratory and cardiac arrest, then the CPR process must be initiated. CPR measures must be taken until they are deemed ineffective and abandoned or stable circulation returns.

## Conclusions

Therefore, we believe the following: (1) The successful HRR depends on the timeliness of the administration of treatment. When administered as soon as possible, CPR can improve the successful HRR. (2) The platinum ten minutes after cardiac arrest means it must be performed by the P-CPR, and other forces are auxiliary. (3) The concept of the heartbeat should be established to guide CPR. (4) Making CPR equipment more readily accessible to the public and improving the public’s awareness of the tools and techniques can improve the successful HRR because rescue strategies will be more likely to be initiated within the platinum ten minutes.

## Data Availability

Not applicable.

## Abbreviations

CPR: Cardiopulmonary resuscitation
HRR: Heartbeat recovery rate
P-CPR: Public cardiopulmonary resuscitation
PCAP: Peri-cardiac arrest period
